# Overcoming the detrimental impact of volumetric muscle loss on segmental fracture healing via the induced membrane technique

**DOI:** 10.1302/2046-3758.146.BJR-2024-0334.R1

**Published:** 2025-06-23

**Authors:** Andrew R. Clark, Michael S. Valerio, Jonathan Kulwatno, Sergey S. Kanovka, Andrew L. Ferrer, Christopher L. Dearth, Stephen M. Goldman

**Affiliations:** 1 Extremity Trauma and Amputation Center of Excellence, Defense Health Agency, Falls Church, Virginia, USA; 2 Department of Surgery, Uniformed Services University of the Health Sciences, Bethesda, Maryland, USA; 3 The Henry M. Jackson Foundation for the Advancement of Military Medicine, Inc., Bethesda, Maryland, USA

**Keywords:** Orthopaedics, Trauma, Fracture healing, fracture healing, rats, bone in the defect, tibial bones, bone mineral density (BMD), composite, tissue injuries, PMMA spacer, Micro-CT, recombinant human bone morphogenetic protein-2

## Abstract

**Aims:**

Open fractures pose a substantial treatment challenge, with adjacent muscle loss being a major complication. The induced membrane (IM) technique has shown promise in treating complicated fractures. The aim of this study is to investigate the impact of adjacent muscle trauma on segmental fracture healing using recombinant human bone morphogenetic protein-2 (rhBMP-2) via the IM technique.

**Methods:**

Skeletally mature male rats (n = 10 to 11 per group) underwent unilateral 3 mm segmental bone defects (SBD) of the tibial diaphysis or a composite tissue injury (CTI), which included a SBD along with volumetric muscle loss (VML). A polymethyl methacrylate (PMMA) spacer was formed within the SBD of each rat. After a four-week period, the PMMA spacer was removed, and the defect was treated with a rhBMP2-impregnated collagen sponge. Longitudinal micro-CT (µCT) imaging was conducted at baseline (Day 0) and at weeks 2, 4, 8, and 12 post-spacer removal to monitor fracture healing progress. At the 12-week postoperative mark, a comprehensive analysis was conducted, including endpoint µCT analysis, evaluation of neuromuscular function, tibia torsional testing, and histological examination.

**Results:**

Longitudinal µCT scans revealed no differences in bone formation or bone mineral density (BMD) at any timepoint between the SBD and CTI groups. High-resolution µCT analysis at the endpoint also showed no variations in bone quality. Torsion testing confirmed that VML did not affect bone strength. Notably, CTI animals exhibited an irreversible reduction in muscle mass and neuromuscular function, which was not observed in the SBD group.

**Conclusion:**

Introducing the additional challenge of VML alongside SBD did not hinder the effectiveness of the induced membrane technique in healing a critical-sized defect.

Cite this article: *Bone Joint Res* 2025;14(6):568–577.

## Article focus

- Volumetric muscle loss (VML) is a major complication in open fractures: it hinders the healing process by disrupting communication between tissues, increasing inflammation, and hindering bone regeneration. This makes treating fractures with VML a substantial challenge.
- The induced membrane (IM) technique is a promising treatment for open fractures. This surgical procedure creates a membrane around the fracture site and delays bone healing with a spacer. We hypothesized that this approach may mitigate the negative effects of VML on fracture healing by providing a better environment for bone growth later.

## Key messages

- The IM technique is effective in treating critical-sized defects in rat tibiae, with or without VML in the surrounding muscle.
- The IM technique appears to mitigate the negative effects of VML on fracture healing, possibly by delaying the start of fracture healing and/or creating an osteopermissive microenvironment.
- VML injuries resulted in reduced muscle weight and strength, but neither the presence of the bone defect nor the IM procedure itself caused any lasting damage to the adjacent muscle.

## Strengths and limitations

- Investigates the effectiveness of the IM technique for treating fractures with VML using a comprehensive approach, to evaluate the impact of VML on bone healing at multiple scales.
- Further research is required to define the precise mechanisms by which the IM technique counteracts the negative effects of VML.
- Sex was not investigated as a biological variable in this study, and thus it is unknown if the results are similar for female rats.

## Introduction

Open fractures, occurring at a rate of approximately 30.7 per 100,000 individuals annually, pose a considerable challenge.^1^ These fractures are often caused by high-energy insults (e.g. motor vehicle accidents, industrial accidents, gunshot wounds, blast exposure) and often involve damage to surrounding tissues such as skin, muscle, and tendons, classifying them as composite tissue injuries (CTI). These multitissue traumas result in significant disability and are detrimental to the patient’s quality of life.^2^

Healthy skeletal muscle plays a well-established role in promoting bone healing by providing stem cells, growth factors, and a robust vascular network.^3^ However, volumetric muscle loss (VML), frequently seen in open long bone fractures, disrupts this beneficial communication between muscle and bone, expanding the injury zone. These comorbid injuries exacerbate inflammation, increase infection risk, and substantially hinder fracture healing, presenting a significant obstacle in the treatment of segmental fractures.^4,5^ Consequently, VML is a major risk factor for fracture nonunion.^6,7^ The complexity of composite injuries involving VML necessitates surgical intervention to salvage the affected limb, and the optimal treatment strategy for such cases remains a topic of debate among orthopaedic surgeons.^8^

The induced membrane (IM) technique, also known as the Masquelet technique, offers a promising approach for managing these complex injuries.^9-11^ This technique involves open reduction and internal fixation, followed by placement of a temporary polymethylmethacrylate (PMMA) spacer within the segmental bone defect (SBD), which often incorporates antibiotics for infection control.^12^ After a period of four to 12 weeks, a second surgery carefully removes the spacer to preserve the surrounding membrane. Subsequently, osteoinductive therapies, such as autograft bone or rhBMP2, are introduced into the membrane to facilitate fracture healing.^13^ The exact mechanisms underlying the IM technique’s effectiveness are still under investigation, but evidence suggests that the membrane formed around the spacer promotes bone regeneration by supplying a vascular network and growth factors to the defect site.^12^ Moreover, the IM technique is believed to create a protected environment that shields the bone regenerate from resorption and infiltration of unwanted fibrous tissue.^14^ This study aimed to evaluate the efficacy of the IM technique in treating critical-sized defects in rat tibiae, with and without VML in the adjacent tibialis anterior (TA) muscle. The hypothesis was that the IM technique would mitigate the negative effects of VML on fracture healing.

## Methods

### Animals

This study was approved by our local Institutional Animal Care and Use Committee and conducted in accordance with the Animal Welfare Act and the principles outlined in the Guide for the Care and Use of Laboratory Animals.^15^ An ARRIVE checklist is included in the Supplementary Material to demonstrate adherence to the ARRIVE guidelines. All animals were housed in facilities accredited by the Association for Assessment and Accreditation of Laboratory Animal Care International. They were maintained on a 12-hour light/dark cycle and provided with access to food and water ad libitum. As prescribed by an a priori power analysis, a total of 22 male Lewis rats, aged ten to 12 weeks and sourced from Charles River Laboratories (USA), were randomly allocated into either a control group (SBD) or an experimental group (CTI). Unfortunately, it was necessary to euthanize one rat due to a hardware malfunction, resulting in sample sizes of n = 10 to 11 per group. To maintain anonymity, the investigators were only provided with non-descriptive animal identification numbers for all subsequent data collection. Due to financial constraints and introduction of variation due to the inability to match age and size between sexes in adult Lewis rats, we have limited this study to include only males, which represents a large majority of the USA active duty force.

### Surgical procedures

**Fig. 1:**
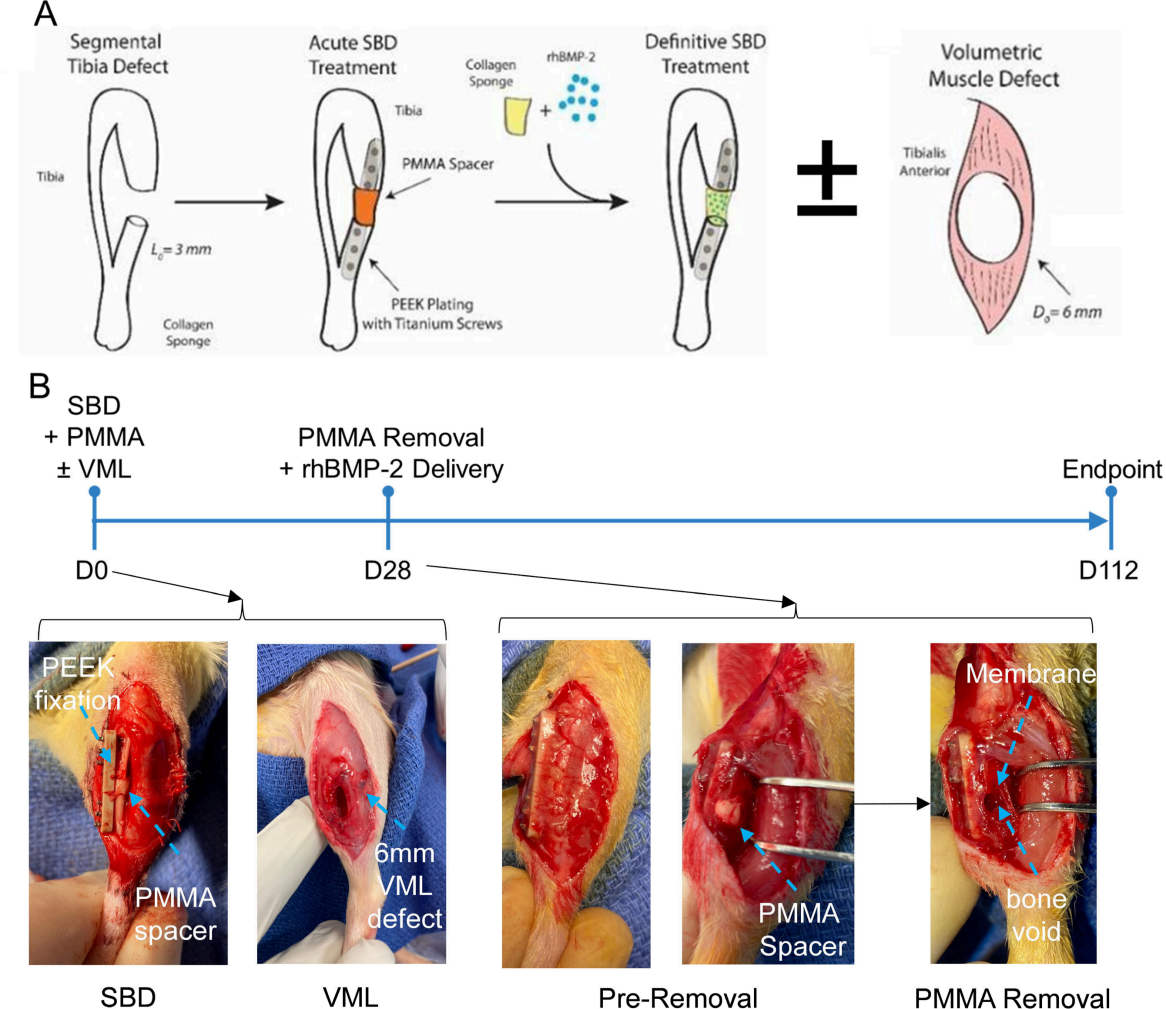
Induced membrane (IM) technique for segmental bone defects (SBD) with and without volumetric muscle loss (VML). a) Schematic of the surgical steps involved in the IM technique for both SBD and SBD+VML. b) Timeline of the injury and surgical phases of the IM technique. SBDs are fixed with a polyether ether ketone (PEEK) plate and polymethyl methacrylate (PMMA) spacer immediately after injury. The spacer is removed four weeks later, revealing the induced membrane and bone void. rhBMP-2, recombinant human bone morphogenetic protein-2.

As depicted in Figure 1, animals received unilateral SBD or CTI (i.e. SBD + VML) injuries in the left hindlimb. Prior to surgery, animals received a dose of Ethiqa XR (Fidelis Animal Health, 0.65 mg/kg, S.C.; USA) for analgesia. Animals were anaesthetized with isoflurane, using 3% to 5% in oxygen for induction and 1% to 3% in oxygen for maintenance. A lateral incision was made to expose the tibialis anterior (TA) muscle by dissecting through the skin and fascia. In the CTI group, VML was induced by excising a 6 mm full-thickness biopsy from the middle third of the muscle belly.^16^ Subsequently, a modular PEEK fixation plate (RISystem, Switzerland; RIS.602.110) was affixed to the tibia using six miniature titanium screws (RISystem, RIS.402.120). A 3 mm full-thickness segmental bone defect was created in the diaphysis using a Gigli saw. A polymethyl methacrylate (PMMA; Zimmer Biomet, USA; 110035368) spacer was moulded inside the defect (Figure 1b), followed by closure of the wound. After four weeks, the rats underwent a second procedure to replace the PMMA spacer with a collagen sponge (Advanced BioMatrix, USA) containing 2 µg of rhBMP2 (R&D Systems, USA). Animals were euthanized 12 weeks after the revision surgery via intracardiac delivery of a sodium pentobarbital solution (Euthasol; Vibrac Corporation, USA) while under anaesthesia. Soft-tissues were excised, weighted, and snap-frozen in optimal cutting temperature (OCT) compound using liquid nitrogen-cooled isopentane for subsequent histological examination. Tibiae were either frozen for subsequent torsion testing or fixed in 10% neutral buffered formalin for ex vivo micro-CT (µCT) scanning before paraffin embedding for histological analysis.

### Neuromuscular functional assessments

Animals were lightly anaesthetized with isoflurane (1% to 3%) and provided thermal support using a heated table throughout the procedure. Neuromuscular function was assessed using a dual-mode muscle lever system (Model 305 C; Aurora Scientific Inc, Canada). The foot was secured to a force transducer with the ankle set at a 90° angle. The tendon of the extensor digitorum longus was exposed and incised. The common peroneal nerve was stimulated using subcutaneous needle electrodes. The current required to induce maximum tetanic contractions (150 Hz, 0.1 ms pulse width, 400 ms train) was determined experimentally for each rat and maintained for the duration of the procedure. Isometric torque about the ankle joint was then measured at a range of stimulation frequencies (10 to 200 Hz).

### In vivo µCT imaging and analysis

Micro-CT imaging was performed on anaesthetized animals (isoflurane 1% to 3%) following injury. Scans were obtained at five timepoints: immediately after PMMA removal (0 timepoint), and then at two, four, eight, and 12 weeks post-surgery. The Siemens Inveon Preclinical Scanner (Erlangen, Germany) was used for imaging, with settings of 80 kV, 500 µA, and 1,000 ms exposure for a 360° rotation scan. The scan generated 515 projection images for reconstruction which employed the Inveon Acquisition Workplace (IAW; Siemens, Germany) using a pre-established Feldkamp cone beam algorithm. Beam hardening and Hounsfield Unit corrections were applied. The reconstructed image size was 1,536 × 1,536 × 1,074 at a voxel size of 55.8 µm. For analysis, reconstructed scans underwent reorientation and segmentation using SkyScan DataViewer software (Bruker, USA). Reoriented scans were then resliced in the coronal plane for analysis using a predefined region of interest (ROI) applied to each image. CTAn analysis software (Version 1.15.4.0; Bruker) was used to segment the ROI between the pins within the images. Thresholding at 95 to 255 was employed for this segmentation to calculate bone mineral density (BMD) and bone volume (BV). Representative 2D scan images were captured from the same location at each timepoint (post-removal, two weeks, four weeks, eight weeks, and 12 weeks).

### Ex vivo µCT imaging and analysis

At the terminal endpoints, soft-tissues were excised, and tibiae were immersed in 10% neutral buffered formalin for a period of seven days. Following fixation, tibiae were washed and stored in phosphate-buffered saline. Subsequently, both proximal and distal segments of the tibia were trimmed, leaving the tibial diaphysis region with the fixation plate and screws intact for scanning. The Bruker SkyScan 1278 ex vivo scanner with a 0.5 mm Al filter was used for imaging. Scanning parameters included 70 kV X-ray energy, 112 µA current, 300 ms integration time, and a voxel size of 6 µm. Reconstruction of the scans employed a smoothing value of 3, ring artifact reduction of 6, and beam hardening correction of 20%. DataViewer software (Version 1.5.2.4, Bruker) ensured consistent reconstruction orientation in terms of angles and rotation, focusing on a ROI within the coronal plane. Subsequently, CTAn software (Version 1.15.4.0, Bruker) was used to analyze resliced coronal sections. The ROI was defined as the standardized region between the last proximal screw and the first distal screw, encompassing 50 slices across all samples. Quantitative analysis of the ROI included BMD measurements using a calibrated phantom and bone morphometry parameters including bone volume (BV), bone surface (BS), object number (Ob.N), degree of anisotropy (DA), fractal dimension (FD), structure thickness (St.Th), and structure separation (St.Sp). Fracture union was determined if there was any amount of continuous bone between the proximal and distal regions of the defect.

### Torsion testing

Following careful removal of their connecting fibula and fixation plates, tibiae were embedded in Field’s metal (Thermo Fisher Scientific, USA) and subjected to torsional testing until failure at a rate of 1°/s using an electrodynamic torsion test machine (560 series, TestResources, USA). The ultimate torque value was recorded for each sample. An ultimate torque of 0 N·m was assigned to samples that were unable to be tested due to failure to achieve fracture union.

### Histology

Samples were sectioned at a thickness of 7 µm. Haematoxylin and eosin (H&E) and Safranin-O staining were performed using standard histological staining methodology (Histoserv, Inc, USA). Picrosirius red (PSR) staining was performed with PSR kit (Abcam, UK; ab150681) per the manufacturer’s protocol. Slides were sealed with Micromount Mounting Medium (Leica, Germany; 3801730). All slides were imaged at ×10 magnification with an Axio Scan Z1 (Zeiss, Germany).

### Statistical analysis

Unless explicitly specified, all data are presented as mean values accompanied by their SDs. Study outcomes were analyzed by analysis of variance (ANOVA) with Holm-Šídák post-hoc testing, using GraphPad Prism 10.2.0 (GraphPad Software, USA). Statistical significance was achieved at an α of 0.05.

## Results

**Fig. 2:**
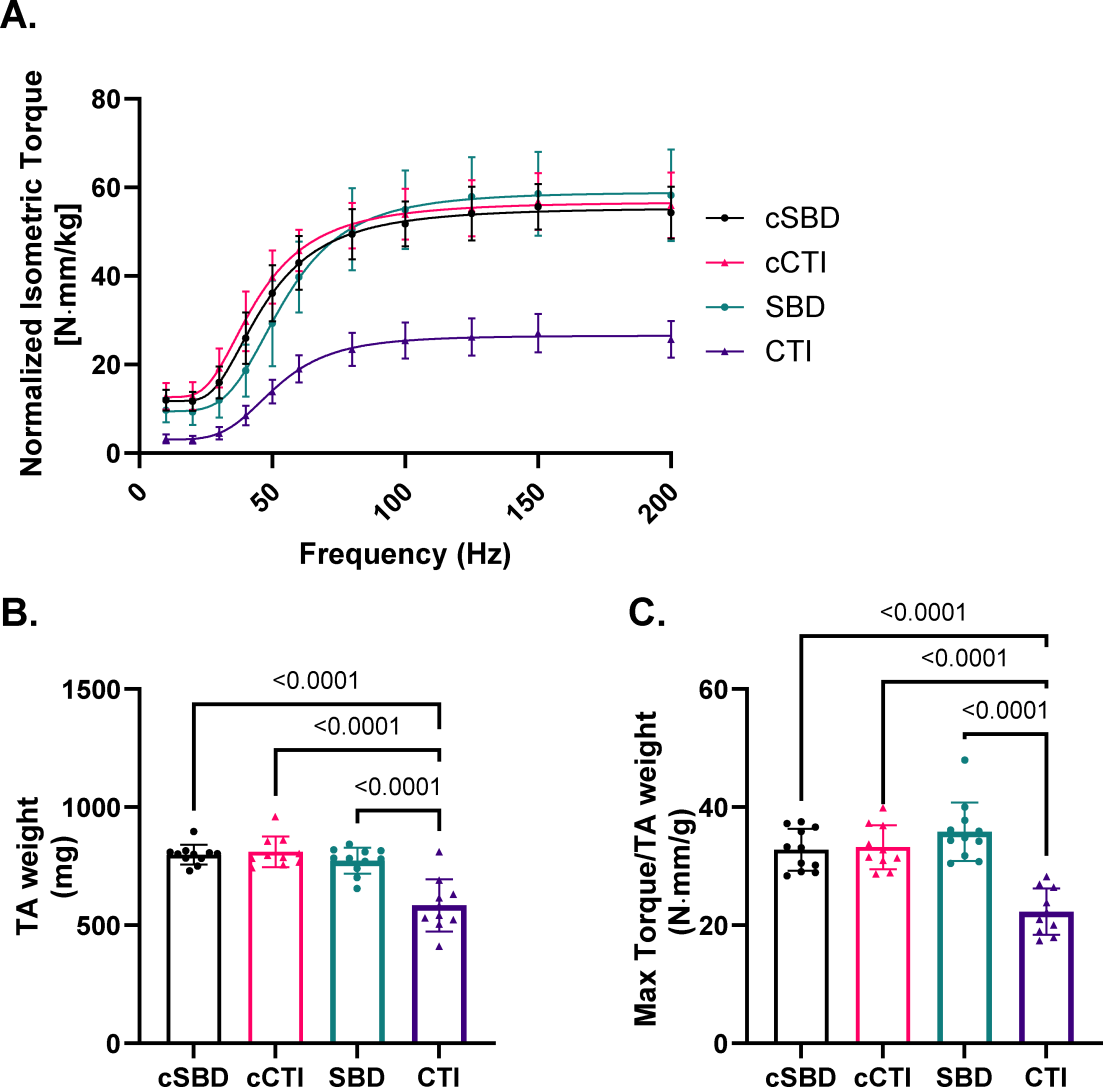
Muscle function and mass compared after segmental bone defect (SBD) and composite tissue injuries (CTIs). a) Neuromuscular torque production, presented as normalized torque per body weight, in response to increasing stimulation frequency. b) Tibialis anterior (TA) muscle weights from SBD, CTI, and their respective contralateral limbs (cSBD, cCTI) at 12 weeks post-injury. c) Maximum torque normalized to TA muscle weight, measured from limbs harvested 12 weeks post-injury. Data were analyzed using analysis of variance followed by Holm-Šídák post hoc tests. Data are represented as means with error bars representing SDs.

Neural evoked functional testing (Figure 2a) revealed a significant decrease in isometric torque generation by the injured limb in the CTI group when compared with both their healthy contralateral limbs and the affected limb of the SBD group (p < 0.001). Furthermore, the TA muscles of the CTI group exhibited a notable reduction in mass (Figure 2b) compared with both their contralateral controls (-226 mg, p < 0.001) and the affected TA muscles from the SBD group (-189 mg, p < 0.001). After adjusting for this difference in muscle size, the injured muscles from the CTI group showed significantly reduced strength relative to their unaffected contralateral limbs (-10.91 N·mm/g, p < 0.001) and the affected limb of the SBD group (-13.55 N·mm/g, p < 0.001). Conversely, the affected limbs from the SBD group demonstrated no meaningful decline in either TA muscle weight (-25.35 mg, p = 0.656) or maximum specific strength (+3.047 N·mm/g, p = 0.243) compared with their contralateral controls (Figure 2c).

**Fig. 3:**
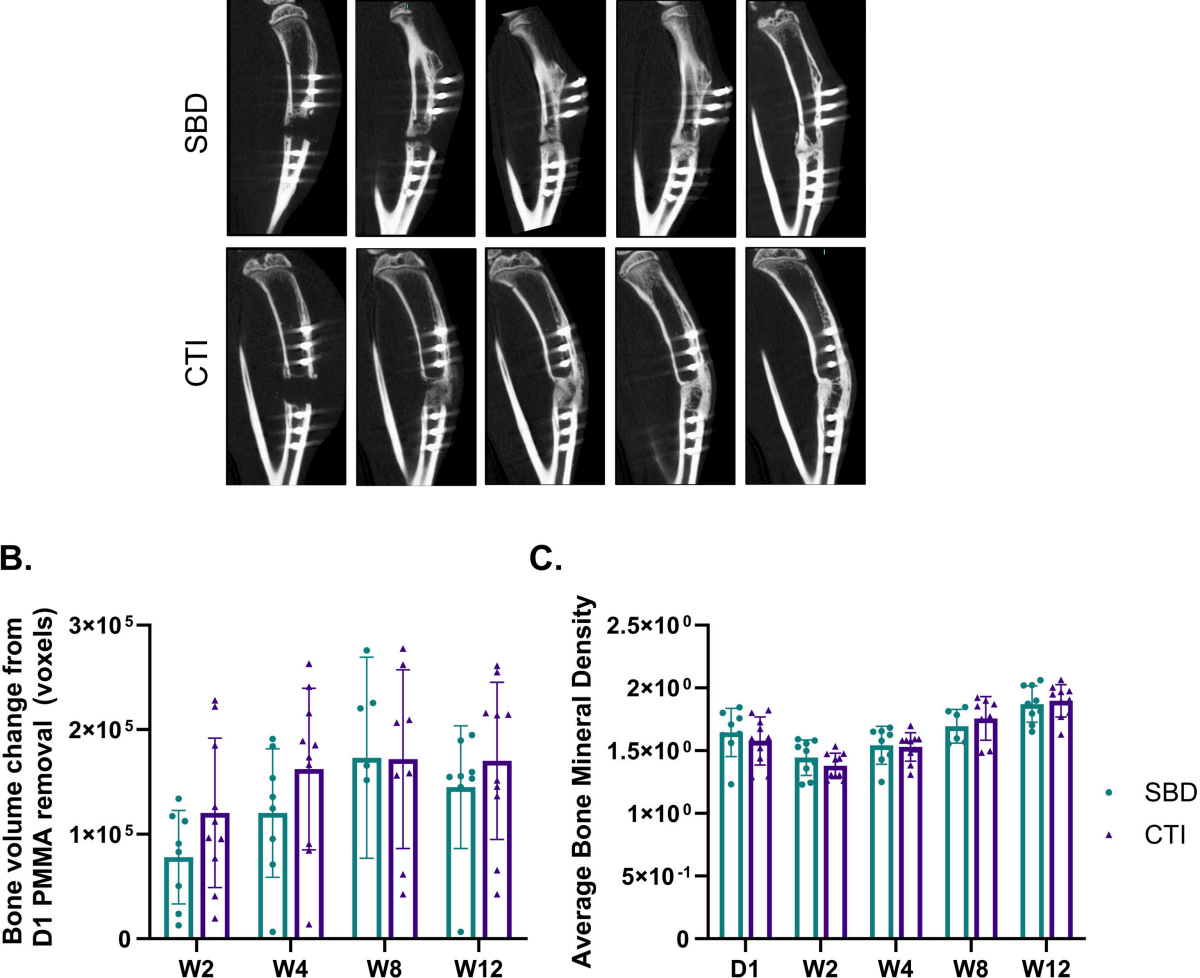
Longitudinal analysis of fracture healing in segmental bone defect (SBD) versus composite tissue injuries (CTI) using micro-CT (μCT). a) Representative μCT scans of SBD and CTI injuries at zero, two, four, eight, and 12 weeks post-injury. b) Changes in bone volume (BV) over the 12-week healing period for SBD and CTIs, following PMMA spacer removal. c) Mean bone mineral density (BMD) throughout the healing period.

Longitudinal µCT scans (Figure 3a) were taken of the injured limbs at regular intervals (0, 2, 4, 8, and 12 weeks) after the removal of the PMMA spacer. Analysis of bone morphometry indicated no disparities in BV (Figure 3b) or BMD (Figure 3c) between SBD and CTI injuries at any of the timepoints. Interestingly, although there were no disparities between the groups, BV demonstrated an increase at weeks 4 and 8 post-injury, leveling off by week 8. Moreover, BMD results show a drop in BMD from one day post-injury to week 2, followed by a steady increase in BMD each week through week 12.

**Fig. 4:**
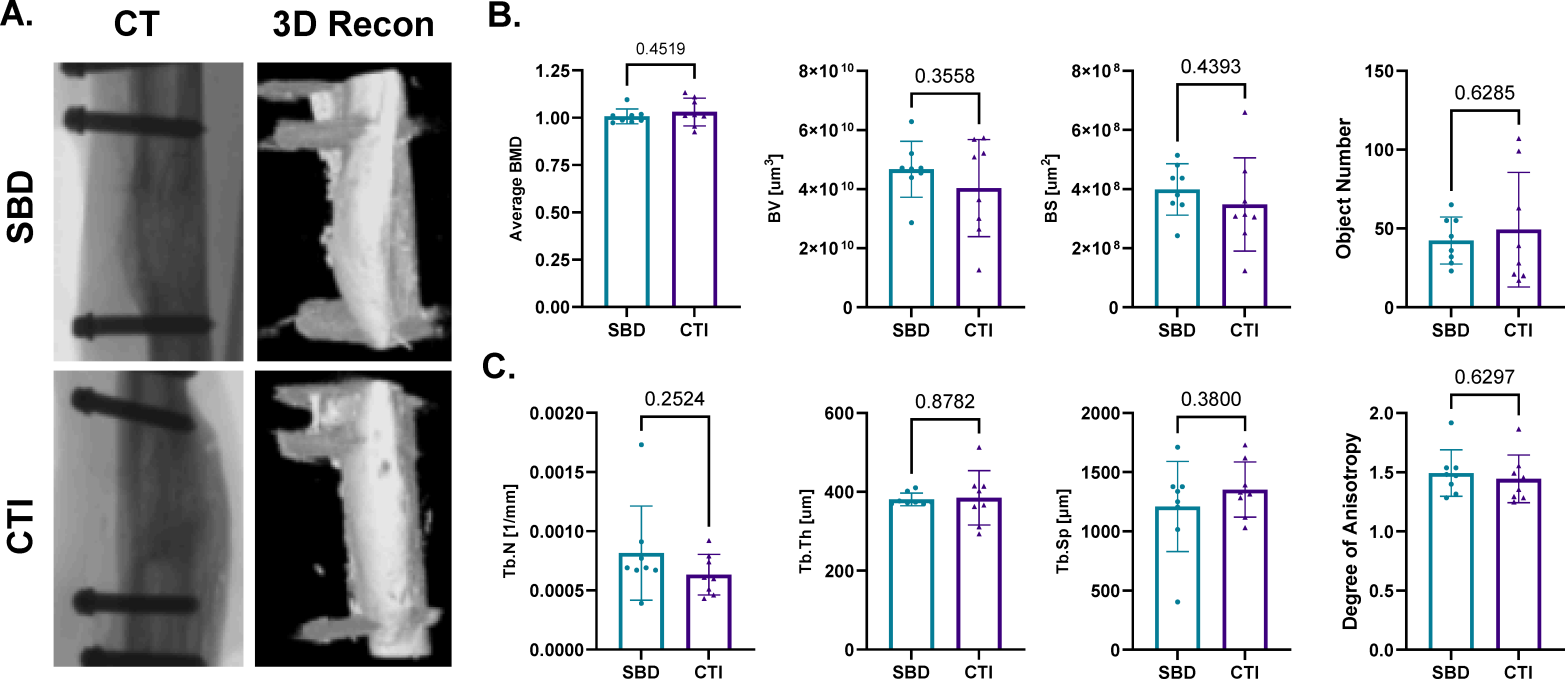
Ex vivo analysis of fracture healing in segmental bone defect (SBD) vs composite tissue injuries (CTIs) at 12 weeks using micro-CT (μCT). a) Representative μCT scans (X-ray and 3D reconstruction) of SBD and CTIs at 12 weeks post-injury. b) Changes in bone mineral density (BMD), bone volume (BV), bone surface (BS), and object number (Obj.N) for SBD and CTI injuries over the 12-week healing period. c) Analysis of trabecular bone structure parameters (trabecular number (Tb.N), trabecular thickness (Tb.Th), trabecular spacing (Tb.S), and degree of anisotropy) at the 12-week endpoint.

Upon completion of the study, high-resolution µCT scans were performed on isolated bones ex vivo to evaluate the microarchitecture of the newly formed bone in the defect region (Figure 4) along with bone morphometry results. BV and mean BMD remained the equivalent (p = 0.356 and p = 0.452, respectively). Additionally, there were no differences in BS (p = 0.439), trabecular number (Tb.N; p = 0.252), trabecular spacing (Tb.S; p = 0.380), trabecular thickness (Tb.Th; p = 0.878), Ob.N (p = 0.152), or DA (p = 0.630).

**Fig. 5:**
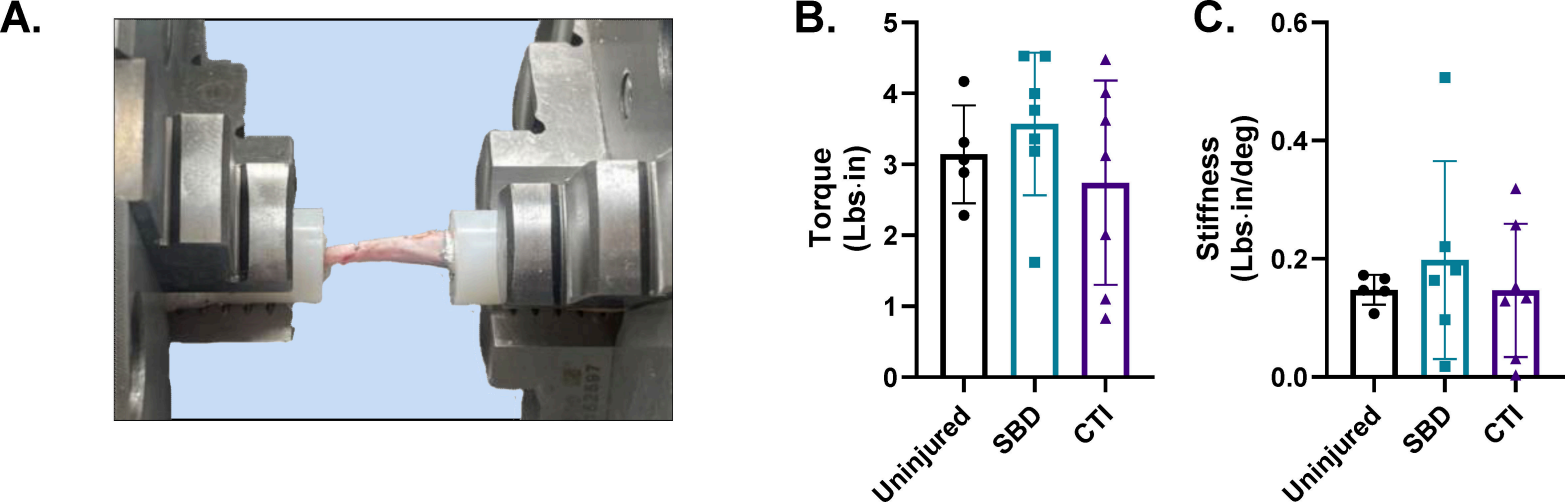
Biomechanical testing of segmental bone defect (SBD) and composite tissue injury (CTI) tibial bones. a) Schematic of the torsion apparatus used to evaluate bone strength. b) Maximum torque at failure (lbs·in). c) Stiffness calculated as torque divided by angle at fracture (lbs·in/deg). Data are represented as mean (SD) of n = four to seven biological replicates.

For each SBD and CTI group, two of the eight bones achieved complete union. To assess the functionality of the healed tibiae, we performed torsional testing to failure. Both the SBD and CTI groups had similar failure torque and stiffness to uninjured tibiae (p = 0.706 and p = 0.223, respectively) (Figure 5).

**Fig. 6:**
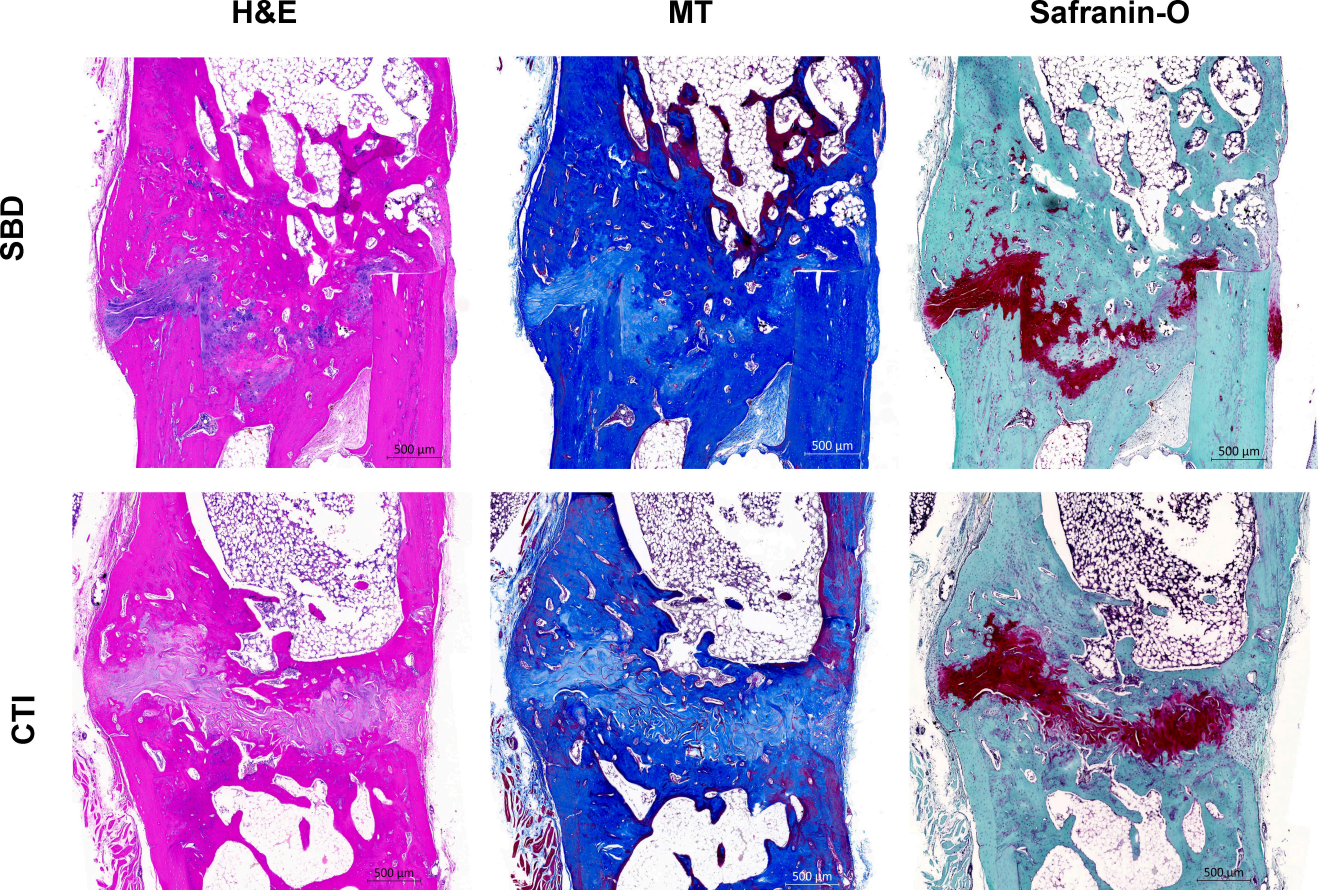
Histological evaluation of fracture healing in segmental bone defect (SBD) versus composite tissue injuries (CTIs). a) Haematoxylin and eosin (H&E) staining to assess cellular infiltration and tissue morphology. b) Safranin O/Fast Green staining to evaluate woven bone formation in the fracture callus. c) Mason’s Trichrome (MT) staining to assess mineralized tissue content and collagen fibre orientation.

As depicted in Figure 6, there were no marked differences in the histological presentation of the SBD and CTI groups at 12 weeks post-injury as H&E, Masson’s trichrome, and Picrosirius red (PSR) staining revealed comparable levels of cellularity and woven bone across both groups.

## Discussion

Our study sought to investigate the impact of the IM technique on segmental fractures exacerbated by concomitant ipsilateral VML injury. We hypothesized that the IM technique could promote fracture healing by delaying rhBMP2 treatment until the VML injury had matured. Our findings support this premise, indicating that the IM technique can facilitate rhBMP-2 mediated healing of a critical-sized bone defect complicated by adjacent VML injury, a scenario where immediate rhBMP-2 treatment has previously proven ineffective.^4,5^ While the precise mechanisms through which the IM technique alleviates the adverse effects of VML on fracture healing remain unclear, several potential mechanisms might contribute. The membrane that forms around the PMMA spacer during the first stage of the IM technique has been shown to release important growth factors and is well vascularized.^17^ These secreted growth factors could create an osteogenic environment sufficient to overcome the negative effects of injured muscle. Moreover, the vascularity of the IM may compensate for the loss of vascular support otherwise attributable to the loss of muscle coverage. Alternatively, the IM may act as a physical barrier, preventing negative inflammatory signals from the injured muscle from reaching the fracture.^7,18^ Additionally, delaying the start of fracture healing may also play a role, as acute inflammation from VML may have a more deleterious impact on fracture healing compared with chronic VML inflammation.^19^

Besides union as the primary outcome measure, we also examined fracture healing over time and observed no differences in the rate of bone healing between the CTI and SBD only groups over the 12-week period. Furthermore, there were no differences in bone ultrastructure or torsional strength and stiffness between the groups at the 12-week timepoint. These findings suggest minimal to no impact of VML on segmental fracture healing when the IM technique is used. These results are noteworthy, as existing literature indicates that VML negatively affects endogenous fracture healing and BMP2 treatment efficacy delivery acutely after injury.^4,20-23^ Moreover, the deleterious impact of muscle damage on fracture healing is clinically observed and is a major consideration in the Gustilo-Anderson classification scale.^24,25^ The ability of the IM technique to overcome the negative effects of muscle damage on fracture healing could explain its efficacy with open fractures.^26-29^

In addition to examining fractures, we evaluated muscle damage and repair at the end of the study. TAs that received VML were lower in weight and exhibited reduced neuromuscular strength, as expected.^16^ However, the SBD procedure did not cause any noticeable lasting damage to the adjacent TA.

Previous research has demonstrated that VML injuries respond to minced muscle graft (MMG) obtained from a donor site.^30,31^ In these cases, it was shown that inflammation caused by VML is blunted. It is conceivable that in future studies, use of MMG to treat VML, used with the IM technique in the presence of SBD, may reduce the time needed for spacing, allowing a more rapid recovery. To that end, as mentioned previously, use of global anti-inflammatories in place of MMG also reduced the inflammatory insult of VML and actually rescued VML-induced nonunion of fractures in an acute setting.^18^ These observations, combined with our findings, may lead to a rapid healing paradigm for complex injuries that are otherwise prone to failure.

There are several limitations of this work. The study only used male rats, which tend to exhibit more robust healing than females.^32^ It is uncertain how different hormone levels and other sex differences may affect the findings of this work. However, if the IM technique’s efficacy is due to increased growth factor/vascularization from the IM, physical barrier from surrounding negative inflammation, or delaying bone regeneration from acute VML inflammation, then these mechanisms would provide at least some benefit in females. Another limitation of this work is the difference in scale of this rat injury compared with those seen in humans. While this 3 mm segmental bone defect is a critical-sized defect in a rat tibia, it does not fully replicate the magnitude of a critical-sized defect in humans which is 2.5 cm or more.^33^ In addition, the IM itself is thinner in a rat than that which forms in a human. It is currently unknown what other differences may exist between the IMs of the two species.

In conclusion, our results suggest that the IM technique mitigates the negative impacts of VML on rhBMP2 mediated healing of segmental fractures and adds to the evidence supporting its use in clinical practice for challenging fractures.

## Data Availability

The data that support the findings for this study are available to other researchers from the corresponding author upon reasonable request.
